# Analytical Comparison of an In-House FARR Fluorescence Immunoassay with Commercial Analytical Assays for the Detection of Anti-Double-Stranded DNA Antibodies

**DOI:** 10.3390/diagnostics16162612

**Published:** 2026-08-18

**Authors:** Manca Ogrič, Saša Čučnik, Katja Lakota

**Affiliations:** 1 Department of Rheumatology, University Medical Centre Ljubljana, 1000 Ljubljana, Slovenia; sasa.cucnik@kclj.si (S.Č.); katja.lakota@kclj.si (K.L.); 2 Faculty of Pharmacy, University of Ljubljana, 1000 Ljubljana, Slovenia; 3 The Faculty of Mathematics, Natural Sciences and Information Technologies (FAMNIT), University of Primorska, 6000 Koper, Slovenia

**Keywords:** anti-dsDNA antibodies, systemic lupus erythematosus, Farr-FIA

## Abstract

**Background/Objectives**: Anti-double-stranded DNA antibodies (anti-dsDNA) are a specific biomarker for systemic lupus erythematosus (SLE). We aimed to analytically compare the in-house Farr fluorescence immunoassay (Farr-FIA), developed based on classical Farr-RIA, for the detection of anti-dsDNA with commercially available assays, including a chemiluminescence assay (CLIA) and the Crithidia luciliae immunofluorescence test (CLIFT). Additionally, we evaluated analytical agreement for diagnostic combinations of these methods. **Methods**: We analyzed 182 samples from patients at the Department of Rheumatology, UMC Ljubljana, using Farr-FIA, CLIFT (Immuno Concepts—CLIFT 1 and Inova Diagnostics—CLIFT 2), and the QUANTA Flash dsDNA CLIA on the BIO-FLASH analyzer (Inova Diagnostics). Agreement between methods and diagnostic combinations was assessed using Cohen’s kappa. **Results**: Overall agreement between methods using manufacturer determined cut-offs ranged from 78.6% to 91.8% (κ = 0.582–0.836). The in-house Farr-FIA showed 78.6–86.8% comparability with commercial assays (κ = 0.582–0.730). Analytical agreement between combinations of two methods ranged from κ = 0.788 to 1.000, indicating moderate to almost perfect agreement. Our routine combination of CLIFT 1 followed by Farr-FIA showed high agreement (κ = 0.788–0.938) with other two-step approaches. Regardless of initial screening (CLIFT 1, CLIFT 2, or CLIA), applying Farr-FIA as a confirmatory method yielded highly consistent final classifications (97.3–100% agreement). However, the screening methods differed in the proportion of samples requiring confirmatory method (e.g., CLIFT 2 identified more positives requiring Farr-FIA), consequently affecting workflow and costs, although the final diagnostic results remain the same. **Conclusions**: Farr-FIA demonstrated moderate to substantial analytical agreement with established commercial assays (CLIA and CLIFT). Several diagnostic combinations demonstrated high analytical agreement; however, the choice of initial screening method substantially influences the number of samples requiring confirmatory testing and, therefore, affects laboratory workload.

## 1. Introduction

Anti-double-stranded DNA (anti-dsDNA) antibodies, first identified in 1957 [1], remain among the most disease-specific serological markers for systemic lupus erythematosus (SLE) [2]. They are included in all major SLE classification criteria (ACR 1982, revised 1997; SLICC; and the recent EULAR/ACR criteria [3,4]). SLE is a chronic autoimmune disorder of unknown etiology, triggered by a complex interplay of genetic, hormonal, environmental, and immunological factors [5,6].

Numerous studies have shown that anti-dsDNA antibodies are not only excellent diagnostic markers but also indicators of disease activity. Increased levels correlate with disease flares and tissue or organ involvement, particularly lupus nephritis [2,4,7]. Importantly, only high-avidity anti-dsDNA antibodies are clinically relevant for SLE and are considered predictive of nephritis development [4]. These antibodies are highly heterogeneous due to the diversity and polyclonality of the autoimmune response to native DNA [8,9,10]. Therefore, the choice of assay for their detection is critical, and laboratories must understand the analytical characteristics of each method to ensure the detection of clinically significant high-avidity anti-dsDNA antibodies that are associated with disease onset and progression [2,11].

Historically, the Farr radioimmunoassay (RIA) [12] was considered the “gold standard” for identifying clinically important high-avidity antibodies. However, safety concerns, workflow limitations, and regulatory restrictions have led to a shift toward non-isotopic methods, such as the Crithidia luciliae indirect immunofluorescence test (CLIFT) [13] and various solid-phase immunoassays, including enzyme-linked immunosorbent assays (ELISA), multiplex flow immunoassay (MFI), chemiluminescence immunoassays (CLIA), multiplex microarrays, and particle-based multi-analyte technology (PMAT) [14,15,16,17]. Very importantly, the assays differ in the antigens they use: Farr-RIA uses native DNA, CLIFT uses Crithidia luciliae kinetoplast DNA as the antigen, and newer solid-phase methods rely on synthetic DNA or DNA fragments. These differences substantially influence the spectrum of anti-dsDNA antibodies detected. To preserve the high-avidity specificity of the Farr-RIA while improving practicality, Lakota et al. developed a modified fluorescent Farr method (Farr-FIA) and compared its performance to the original Farr-RIA [18].

Despite these advances, controversies remain regarding the optimal methods for screening, diagnosis, disease monitoring, and therapy assessment [2]. For classification purposes, assays must achieve specificity greater than 90% [14]. Harmonization remains challenging due to differences in cut-offs, avidity selection, antigen source, and calibration against imperfect reference materials [17,19].

Current diagnostic algorithms therefore recommend a two-step approach to anti-dsDNA testing that leverages the complementary strengths of different assay platforms. A sensitive solid-phase assay such as ELISA, CLIA, or FEIA is used for initial screening, as these methods efficiently detect a broad spectrum of anti-dsDNA antibodies. This is followed by a highly specific confirmatory assay—typically CLIFT or a Farr-type assay (Farr-RIA or Farr-FIA)—to verify true anti-dsDNA reactivity and identify clinically important high-avidity antibodies. This strategy minimizes false positives, improves overall diagnostic accuracy and maintains sensitivity for active SLE when integrated with clinical assessment [2,11].

The aim of this study was to analytically compare the results of our in-house Farr-FIA with those of commercially available assays: CLIA (QUANTA Flash^®^ dsDNA, Inova Diagnostics Inc., San Diego, CA, USA) and CLIFT (Inova Diagnostics Inc., San Diego, CA, USA, and Immuno Concepts, Sacramento, CA, USA). Additionally, we aimed to evaluate analytical agreement across different diagnostic algorithms combining two methods for anti-dsDNA testing.

## 2. Materials and Methods

### 2.1. Samples

Between January and May 2024, 182 serum samples were collected from patients sent for routine anti-dsDNA testing at the Department of Rheumatology, University Medical Center, Ljubljana. The study included 97 patients with SLE (92% female; median age 47.1; IQR 34.0–56.3). The control group consisted of 85 patients with other rheumatic diseases or without confirmed rheumatic disease (69% female; median age 54.7, IQR 39.4–67.7). The included patients signed an informed consent form (Slovenian Medical Ethics Committee 0120-118/2023-2711-9). All consecutive samples submitted for routine anti-dsDNA testing during the study period were eligible if informed consent was signed. Samples with insufficient volume for testing by all four assays were excluded. No samples were excluded for quality issues.

### 2.2. Methods

The CLIFT assays (Immuno Concepts, Sacramento, CA, USA and Inova Diagnostics Inc., San Diego, CA, USA) were performed according to the manufacturers’ instructions. The QUANTA Flash dsDNA CLIA (Inova Diagnostics Inc., San Diego, CA, USA) was performed on the BIO-FLASH analyzer, and the Farr-FIA was carried out following in-house protocol [18]. A comparison of the diagnostic and analytical features of the methods used is presented in Table 1. All assays were performed independently, and assay operators were blinded to the results of the other anti-dsDNA assays.

#### 2.2.1. CLIFT

Crithidia luciliae cells immobilized on glass slides were incubated with patient samples diluted 1:10 in PBS for 30 min at room temperature. After washing with PBS, FITC-conjugated anti-human IgG was added and incubated for an additional 30 min, followed by a second wash. Slides were examined under a fluorescence microscope at 495 nm. Samples positive for anti-dsDNA showed green fluorescence in the kinetoplast region or near the nucleus. Results were read by two independent raters and, in case of discrepancy in the result, a third one read the slides.

#### 2.2.2. CLIA

QUANTA Flash dsDNA reagents were used according to the manufacturer’s instructions on the BIO-FLASH^®^ analyzer (Inova Diagnostics Inc., San Diego, CA, USA). Briefly, magnetic microparticles coated with dsDNA were used to capture anti-dsDNA from the sample. Results are expressed in IU/mL. According to the manufacturer, the threshold for a positive test is 35 IU/mL. Values between 27 and 35 IU/mL are considered equivocal and were analyzed in the positive result group.

#### 2.2.3. Farr-FIA

A detailed description is provided in reference [18]. Briefly, a working solution of dsDNA was prepared by mixing 95 µL borate buffer (0.15 M, pH 8.0) with 5 µL of isolated human dsDNA (whole blood DNA isolation using the QIAamp DNA mini QIAcube kit, Qiagen, Hilden, Germany). This mixture was combined with 5 µL decomplemented serum and incubated at 37 °C for 1 h, and then overnight at 4 °C. After 16–18 h, 100 µL saturated ammonium sulfate was added and incubated at 4 °C for 1 h. Samples were centrifuged at 1800 *g* for 15 min at 4 °C, and 100 µL of the supernatant was separated from the pellet. Ten microliters of each fraction were mixed with 90 µL PicoGreen reagent (1:200 dilution; Quant-iT™ PicoGreen dsDNA Assay Kit, Invitrogen, Thermo Fisher Scientific, Waltham, MA, USA) in a 96-well plate. Fluorescence was measured in the supernatant (S) and precipitate (P) using an Infinite F200 spectrophotometer (Tecan, Männedorf, Switzerland), and binding was expressed as (P − S)/(P + S), representing the ratio of bound to unbound dsDNA–anti-dsDNA complexes [18].

### 2.3. Combination of Methods

In our routine diagnostics, CLIFT 1 is used in the initial screening phase, with positive results subsequently confirmed using a high-avidity assay (Farr-FIA). According to the current recommendations, screening assays with high sensitivity that detect low-, medium-, and high-avidity antibodies include ELISA, FEIA, and CLIA, while assays with high specificity include the Farr-RIA and CLIFT assays.

We compared our diagnostic approach (CLIFT 1 and Farr-FIA) with other recommended testing strategies:

- CLIFT 2 and Farr-FIA;
- CLIA and Farr-FIA;
- CLIA and CLIFT 1;
- CLIA and CLIFT 2.

### 2.4. Statistical Analysis

Agreement between methods and between method combinations was assessed using Cohen’s kappa coefficient and the percentage of agreement (%). Agreement among methods was calculated at the recommended manufacturers’ cut-off values. Correlation between quantitative methods was analyzed using Spearman’s rank coefficient (ρ) for nonparametric data. Statistical significance was set at α = 0.05 (*p* < 0.05 was considered significant). Analyses were performed using Analyse-it (Analyse-it Software Ltd., Leeds, UK) in Excel (Microsoft Corp., Redmond, WA, USA). Heatmaps were prepared with unsupervised clustering of samples and methods with ClustVis [20]. Clustering of both samples and methods/combinations was performed using correlation distance and average linkage.

## 3. Results

### 3.1. Comparison of In-House Farr-FIA with CLIA, CLIFT 1, and CLIFT 2

#### 3.1.1. Positivity of Anti-dsDNA Across Assays and Correlations with SLE Diagnosis

Of the 182 tested samples, all four assays had concordant results in 73.1% of the samples; specifically, 75 (41.2%) were negative and 58 (31.9%) were positive in all four assays. Among the 75 negative samples, 59 were from the control group and 16 from the SLE group. Of the fifty-eight positive samples in all four assays, only three were from the control group.

Among 49 samples (26.9%) that showed discordant results across assays, 17 samples were positive only with both CLIFT 1 and CLIFT 2, and 11 were positive exclusively with CLIFT 2. Four samples were positive with Farr-FIA and both CLIFT assays but negative with CLIA, whereas five samples were positive with CLIA and both CLIFT assays but negative with Farr-FIA. An additional 12 samples showed other patterns of isolated or partial positivity in one or two assays. Among these 49 samples, 26 samples were from patients with SLE and 23 were from the control group.

**Figure 1:**
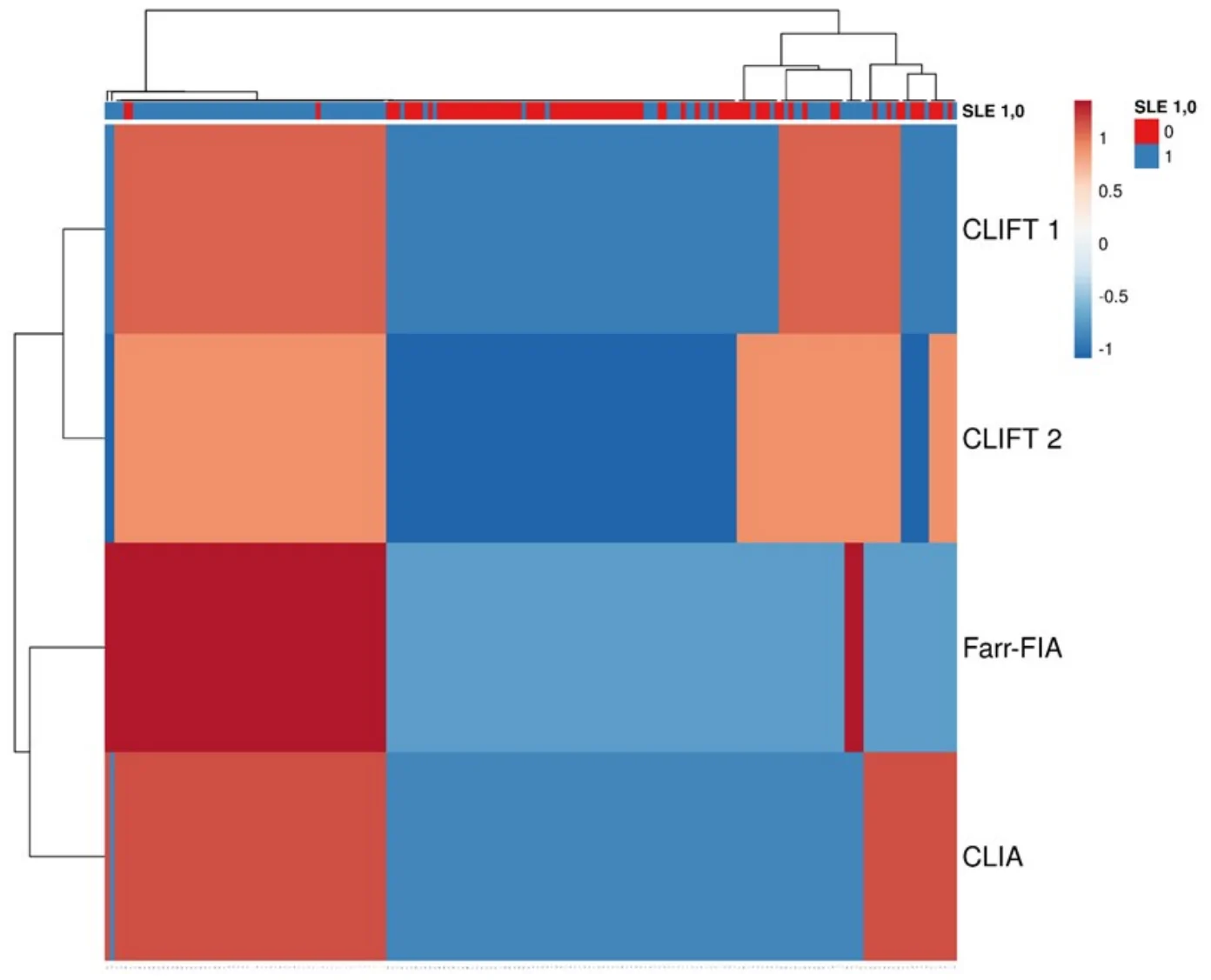
Hierarchical clustering of samples based on anti-dsDNA assay results. Legend: Heatmap with unsupervised hierarchical clustering of individual samples tested by Farr-FIA, CLIA, CLIFT 1, and CLIFT 2. Columns represent individual samples and rows represent anti-dsDNA assays. Samples are annotated according to clinical diagnosis (SLE or control group). Red indicates positive and blue indicates negative assay results. Clustering of both samples and assays was performed using correlation distance and average linkage. The close clustering of CLIFT 1 and CLIFT 2 reflects their high analytical agreement, while the overall heatmap illustrates the heterogeneity of anti-dsDNA positivity patterns across the study cohort.

A heatmap visualization (Figure 1) summarizes the partial separation between SLE patients and control group samples, with areas of concentrated high signal values aligning more frequently with SLE patients’ cases. Distinct assay-specific patterns in the heatmap highlight the differential analytical sensitivity and specificity of individual detection methods. This visualization underscores the heterogeneity of anti-dsDNA responses in SLE and the complementary diagnostic value of combining multiple detection methods.

#### 3.1.2. Overall Agreement Between All Methods

Pairwise distributions of positive and negative results are presented in Table 2. Agreement statistics derived from these data are summarized in Table 3. Overall agreement between the methods ranged from 78.6% to 91.8%, and the corresponding Cohen’s kappa coefficients ranged from 0.582 to 0.836, with both metrics being highest for comparison between CLIFT 1 and CLIFT 2 (Table 3). This is graphically observed also with the unsupervised clustering of methods that discriminates both CLIFT assays from Farr-FIA and CLIA (Figure 1).

#### 3.1.3. Farr-FIA and CLIA

To compare the positive and negative results between CLIA and Farr-FIA, Cohen’s kappa coefficient was calculated to assess qualitative agreement. The obtained Cohen’s kappa value of 0.714 (95% CI: 0.612 to 0.817) indicates substantial agreement. The distribution of positive and negative results for both methods is presented in Table 2.

The Spearman’s rank correlation was used to evaluate the relationship between the two methods. The analysis revealed a statistically significant positive correlation (ρ = 0.67; 95% CI: 0.561–0.756; *p* < 0.0001).

#### 3.1.4. CLIFT 1 and CLIFT 2

When comparing two semi-quantitative indirect immunofluorescence kits from different manufacturers (Immuno Concepts and Inova Diagnostics), both based on Crithidia luciliae cells (CLIFT), the overall agreement according to semi-quantitative intensity scores (0, 1, 2, 3) was 67.0%, with a Cohen’s kappa of 0.509 (95% CI: 0.422–0.597), indicating moderate agreement.

However, when the results were interpreted dichotomously as positive or negative, agreement improved substantially. Overall agreement increased to 91.8%, and Cohen’s kappa rose to 0.836 (95% CI: 0.758–0.915), reflecting almost perfect agreement between the two CLIFT assays (Table 2 and Table 3), a pattern also recognized with hierarchical clustering (Figure 1). This improvement is likely explained by differences in fluorescence intensity grading between the two kits, which affect semi-quantitative categorization but have limited impact on the final positive/negative classification.

### 3.2. Analytical Comparison of Diagnostic Combinations

In the following section, we provide a detailed comparison of our diagnostic algorithm (CLIFT 1 followed by Farr-FIA) with other two-step testing strategies (CLIFT 2 and Farr-FIA, CLIA and Farr-FIA, CLIA and CLIFT 1, and CLIA and CLIFT 2).

#### 3.2.1. Positivity of Anti-dsDNA Across Different Diagnostic Combinations

**Figure 2:**
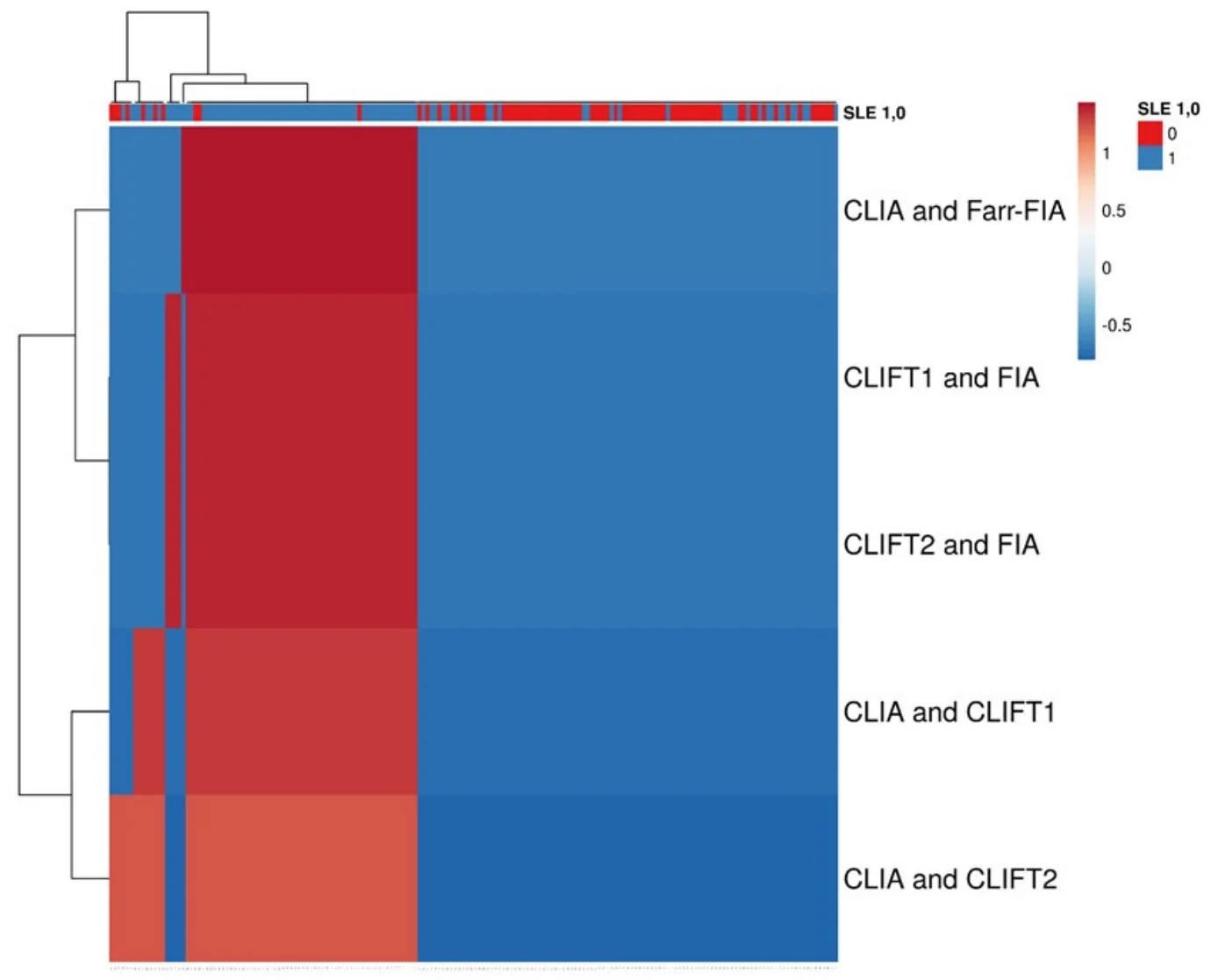
Hierarchical clustering of anti-dsDNA testing algorithms. Legend: Heatmap with unsupervised hierarchical clustering of five anti-dsDNA testing algorithms consisting of two subsequent testing methods: CLIFT 1–Farr-FIA, CLIFT 2–Farr-FIA, CLIA–Farr-FIA, CLIA–CLIFT 1, and CLIA–CLIFT 2. Columns represent individual samples and rows represent testing algorithms. Red indicates a positive final classification, and blue indicates a negative final classification. Clustering of both samples and algorithms was performed using correlation distance and average linkage. The heatmap visualizes the overall similarity between testing strategies and highlights the limited subset of samples responsible for discordant classifications between algorithms.

When analyzing predefined assay combinations, 89.6% (163/182) of the sample results were equal on all five recommended testing combinations for diagnostic testing (CLIFT 1 and Farr-FIA, CLIFT 2 and Farr-FIA, CLIA and Farr-FIA, CLIA and CLIFT 1, and CLIA and CLIFT 2). Among these, 105 of the 182 samples (57.7%) were negative across all five combinations (75 were from the control group and 30 were from patients with SLE). The “positive” intersection included 58 samples (55 SLE, three controls) that were positive in all five recommended testing combinations for diagnostic testing (CLIFT 1 and Farr-FIA, CLIFT 2 and Farr-FIA, CLIA and Farr-FIA, CLIA and CLIFT 1, and CLIA and CLIFT 2). To illustrate these analytical relationships across method combinations and samples, we visualized the data using a heatmap (Figure 2). As expected, algorithms sharing Farr-FIA as the confirmatory step demonstrated the highest agreement, largely reflecting the influence of a common final confirmatory method. A more laboratory-relevant distinction between algorithms was the number of samples requiring confirmatory testing, which differed substantially according to the screening assay used.

A smaller subset of samples showed positivity in only a limited set of combinations (10.4%). Eight samples (five SLE, three controls) were positive exclusively in the combinations CLIA–CLIFT 1 and CLIA–CLIFT 2, while six samples (two SLE, four controls) showed isolated positivity only in CLIA and CLIFT 2. Another group of four SLE samples was positive only in CLIFT 1–Farr-FIA and CLIFT 2–Farr-FIA. One SLE sample was positive solely in the CLIA and Farr-FIA combination.

#### 3.2.2. Overall Agreement Between Diagnostic Combinations

All evaluated combinations and their corresponding Cohen’s kappa coefficients are presented in Table 4.

#### 3.2.3. CLIFT 1 vs. CLIFT 2 in Combination with Farr-FIA

According to our results, the combination of CLIFT 2 with Farr-FIA performed comparably to the standard CLIFT 1–Farr-FIA algorithm in clinical practice (κ = 1.000, Table 4). However, CLIFT 2 detected anti-dsDNA antibodies in 15 additional samples (four SLE and eleven control samples), all of which tested negative according to Farr-FIA. Thus, although CLIFT 2 is suitable as a screening assay when followed by Farr-FIA, its higher false-positive rate must be considered. Overall, 8.2% more samples required confirmatory Farr-FIA testing when CLIFT 2 was used as the initial screening method compared with CLIFT 1, but four SLE patients were missed. However, all four had negative Farr-FIA.

#### 3.2.4. CLIA and CLIFT 1/CLIFT 2 Compared to CLIFT 1 and Farr-FIA

In this diagnostic algorithm, CLIA is used as the initial screening method, detecting both low- and high-avidity anti-dsDNA antibodies. Only CLIA-positive samples are subsequently tested with CLIFT, which effectively excludes low-avidity anti-dsDNA antibodies due to its high-stringency binding conditions. As a result, the final outcome reflects medium- to high-avidity dsDNA positivity.

Agreement between the routine algorithm (CLIFT 1 followed by Farr-FIA) and the alternative CLIA-based algorithms (CLIA followed by CLIFT) was excellent, with Cohen’s kappa values of 0.855 for the CLIA–CLIFT 1 combination and 0.788 for CLIA–CLIFT 2.

When comparing CLIA–CLIFT 1 to the routine approach (CLIFT 1 followed by Farr-FIA), 12 samples were classified differently (four additional positives in CLIFT 1-Farr-FIA—all SLE patients and eight additional positives in CLIA-CLIFT 1; five SLE, three control group). For the CLIA–CLIFT 2 combination, 18 samples differed in classification (four additional positives in CLIFT 1-Farr-FIA—SLE patients and fourteen additional positives in CLIA–CLIFT 2; seven SLE, seven control group) compared with the routine diagnostic testing.

#### 3.2.5. CLIA and Farr-FIA Compared to CLIFT 1 and Farr-FIA

Another possible diagnostic combination is using CLIA in the screening phase followed by Farr-FIA as the confirmatory assay. Cohen’s kappa coefficient for this combination was 0.938 (95% CI: 0.885 to 0.992), and agreement was 97.3%. Overall, discrepancies were observed in five samples.

In one case, the sample tested negative in the routine screening step (CLIFT 1) but was positive according to CLIA (screening step in comparable algorithm); subsequent testing with Farr-FIA confirmed positivity. The remaining four samples were positive according to the routine algorithm (CLIFT 1 and Farr-FIA) but were negative in the CLIA–Farr-FIA algorithm. According to the negative CLIA (a comparable algorithm), these four samples would not have been subjected to Farr-FIA; however, because all samples were evaluated by all methods in this study, Farr-FIA testing revealed that all four were indeed positive. All five of these patients have SLE.

Combinations that included Farr-FIA demonstrated the strongest analytical agreement, forming a distinct cluster characterized by consistently high agreement values, while combinations involving CLIA and CLIFT (CLIA–CLIFT 1 and CLIA–CLIFT 2) showed lower agreement levels, indicating limited agreement within this method combinations.

Both CLIFT 1–Farr-FIA and CLIFT 2–Farr-FIA demonstrated higher analytical agreement than the CLIA–CLIFT combinations, suggesting that FIA-based methods yield outcome patterns that are more comparable to those of the CLIFT assays than to those of CLIA.

## 4. Discussion

Despite the introduction of new high-throughput technologies into daily routine use, traditional assays still offer certain advantages. As reported by Lakota et al. [18], the Farr-FIA is an excellent alternative to the traditional “gold-standard” Farr-RIA for highly specific detection of clinically important high-avidity anti-dsDNA antibodies in patients with SLE, demonstrating strong correlation with the original Farr-RIA assay and superior correlation with SLEDAI. Detailed analytical validation, including assay precision and reproducibility, was also reported in that study. Nevertheless, Farr-FIA remains an in-house assay that uses internally prepared human dsDNA antigen. Assay performance and dsDNA batch consistency are routinely monitored using internal quality control samples and lot-to-lot control samples, allowing for continuous assessment of variability. However, its performance has not been systematically compared with other commercially available diagnostic methods. Such comparisons are essential to evaluate broader applicability, inter-method comparability, and the potential implementation of the Farr-FIA into other laboratory workflows worldwide.

When evaluating agreement across all methods based on qualitative (positive/negative) classification using manufacturer-defined cut-offs, our overall agreement ranged from 78.6% to 91.8%, and Cohen’s kappa values ranged from 0.582 to 0.836, which is consistent with those reported by Infantino et al. [14], who compared eight commercial methods and observed agreement values ranging from 58.8% to 88.8% and kappa coefficients from 0.732 to 0.951. As in their observation, we found the highest agreement and Cohen’s kappa value between CLIFT 1 and CLIFT 2 (91.8% and 0.836), as these are simply variations of the same assay.

Six comparative studies [14,15,21,22,23,24] consistently conclude that no single anti-dsDNA assay performs optimally in every clinical setting, and each method has characteristic strengths and limitations. Our findings are consistent with these observations, as a subset of samples showed discordant results between assays and testing algorithms, likely reflecting the biological heterogeneity of anti-dsDNA antibodies and differences in assay design, antigen source, and antibody avidity recognition. Farr-FIA preferentially detects high-avidity antibodies, whereas CLIA may also detect lower-avidity antibodies because of differences in antigen presentation and binding conditions. This interpretation is supported by the observation that some samples were positive according to CLIA and CLIFT but negative according to Farr-FIA, whereas others were positive according to Farr-FIA and CLIFT but negative according to CLIA. Conversely, CLIFT detects antibodies recognizing native kinetoplast DNA but may not identify all antibody populations detected by solid-phase assays. These methodological differences help explain the variation in analytical performance observed among anti-dsDNA assays. Solid-phase assays such as ELISA and CLIA generally show higher sensitivity, making them suitable for initial screening and for monitoring fluctuations during active SLE or lupus nephritis flares. In contrast, CLIFT and Farr-FIA/RIA assays consistently demonstrate the highest specificity across cohorts, supporting their use as confirmatory methods rather than as primary screening tools.

Studies that directly evaluated assay combinations found that pairing a sensitive screening assay (ELISA or CLIA) with a specific confirmatory assay (CLIFT or Farr-RIA/Farr-FIA) consistently improves overall diagnostic performance, resulting in higher combined sensitivity and specificity than individual methods. This effect is observed across various platforms, including combinations such as ELISA + CLIFT, CLIA + CLIFT, and CLIA/PMAT + CLIFT, which enhance diagnostic discrimination. Overall, the evidence strongly supports an integrated, multi-assay testing approach as the most robust strategy for the accurate detection of clinically meaningful high-avidity anti-dsDNA antibodies [14,15,22]. In our study, all tested combinations of methods (Figure 2) identified 89.6% of samples equally, 57.7% were negative across all method combinations, and 31.9% of samples were positive across all five combinations, with the vast majority originating from the SLE group (55/58).

According to the previous study by Žigon et al. [22], using CLIFT 1 followed by confirmation with Farr-RIA was identified as the optimal diagnostic approach in our clinical setting, primarily because it reduced the number of samples requiring high-specificity testing and shortened turnaround time. In a subsequent study by Lakota et al. [18], Farr-RIA was replaced in our routine diagnostic workflow by the analytically equivalent Farr-FIA. In this study, we reassessed whether CLIFT 1 remains the most appropriate screening assay. Consistent with the findings of Žigon et al., we found that CLIFT 2 (Inova Diagnostics) yielded more positive samples that were negative by Farr-FIA (8.2% of patients), indicating a higher false-positive rate. However, when combined with Farr-FIA, both CLIFT 1 and CLIFT 2 ultimately produced identical final classifications. Agreement between the two diagnostic pathways (CLIFT 1 and Farr-FIA vs. CLIFT 2 and Farr-FIA) was 100%. Expressed as Cohen’s kappa, agreement between different algorithmic combinations ranged from 0.788 to 1.000, indicating moderate to almost perfect agreement.

In line with the current recommendations, alternative diagnostic algorithms using CLIA as the initial screening assay may also be appropriate. Therefore, we evaluated combinations involving CLIA followed by Farr-FIA or CLIFT (either CLIFT 1 or CLIFT 2). Comparisons of these three CLIA-based approaches with the routine CLIFT 1 and Farr-FIA algorithm showed the highest overall agreement among all evaluated combinations (Cohen’s kappa values 0.788–0.938). Differences in positivity rates among other assays (CLIFT 1, CLIFT 2, and CLIA) did not affect the final diagnostic interpretation when Farr-FIA was used as the confirmatory step, and only the time and costs differ a lot between combinations.

Emerging technologies such as particle-based multi-analyte technology (PMAT), multiplex bead assays, and microarray-based platforms are increasingly being used for anti-dsDNA testing. These approaches offer high throughput, automation, and simultaneous assessment of multiple autoantibodies. Recent studies have demonstrated promising analytical performance; however, challenges related to harmonization, clinical validation, and comparability with traditional high-specificity assays remain. Consequently, highly specific methods such as CLIFT and Farr-FIA/RIA assays continue to play an important role as confirmatory tests in diagnostic algorithms.

This study has several limitations. First, no therapy-naïve patients and no samples collected at disease onset were available. Furthermore, detailed clinical data, including disease activity scores (e.g., SLEDAI), organ involvement, and treatment status, were not systematically available for all patients. Consequently, diagnostic sensitivity and specificity, as well as associations with disease activity or specific clinical manifestations, could not be evaluated. However, these performance characteristics of Farr-FIA have been reported previously by Lakota et al. [18], who demonstrated excellent agreement with Farr-RIA and a strong association with disease activity. Second, the impact of the study would be strengthened by including a broader range of commercially available assays, particularly given the diversity of platforms currently used worldwide.

Despite these limitations, our findings emphasize that certain methods—based on available evidence and analytical characteristics—remain the most appropriate first-line tools for detecting anti-dsDNA antibodies. However, these assays are not universally implemented, partly due to the wide availability of commercial methods that are easier to automate and integrate into routine workflows. Laboratories should therefore be cautious when selecting anti-dsDNA diagnostic algorithms, as methodological differences may influence clinical interpretation.

## 5. Conclusions

This study highlights the value of our high-specificity Farr-FIA assay, which showed that its results are comparable to those obtained with CLIA and CLIFT 1/2, demonstrating moderate to almost perfect agreement.

Alternative diagnostic algorithms using CLIA followed by CLIFT or Farr-FIA demonstrated excellent analytical agreement with our routine CLIFT 1–Farr-FIA combination; however, clinical equivalence cannot be concluded from analytical agreement alone. These findings indicate that several method combinations are analytically comparable; however, incorporating a high-specificity confirmatory test such as Farr-FIA remains essential for ensuring clinically meaningful and reproducible anti-dsDNA results in patients with SLE.

## Figures and Tables

**Table 1:** Diagnostic and analytical features of methods.

Assay	Manufacturer/ Instrument	dsDNA Antigen	Cut-Off (Manufacturer)	Diagnostic Sensitivity, Specificity (%)	Isotype	Avidity
Farr-FIA	In-house Farr fluorescence immunoassay	Human	0.35 [18]	53, 100 [18] *	IgG, IgM, IgA	High
CLIA	Chemiluminescence assays on BIO-FLASH analyzer (QUANTA FLASH)	Synthetic polynucleotide	35 IU/mL (27–35 gray zone)	49.1, 87.5 **	IgG	Low to high
CLIFT 1	Immuno Concepts	Native kinetoplast of Crithidia luciliae cells	1:10	30.2, 99.0 **	IgG	Medium to high
CLIFT 2	Inova Diagnostics	Native kinetoplast of Crithidia luciliae cells	1:10	79.2, 96.4 [14] *	IgG	Medium to high

Diagnostic sensitivity and specificity values were derived from previously published studies * or manufacturer documentation ** and were not calculated in the present cohort.

**Table 2:** Distribution of positive and negative results between methods: in-house Farr-FIA, CLIA (QUANTA Flash dsDNA), CLIFT 1 (Immuno Concepts), and CLIFT 2 (Inova Diagnostics).

		CLIA (QUANTA Flash dsDNA), Cut-Off 27 IU/mL		CLIFT 1		CLIFT 2	
		Negative	Positive	Negative	Positive	Negative	Positive
**Farr-FIA,** **Cut-Off 0.35**	Negative	98	20	96	22	81	37
	Positive	5	59	2	62	2	62
		**CLIFT 1**		**CLIFT 2**			
		negative	positive	negative	positive		
**CLIA** **(QUANTA Flash dsDNA),** **Cut-Off 27 IU/mL**	Negative	85	18	76	27		
	Positive	13	66	7	72		
		**CLIFT 2**					
		negative	positive				
**CLIFT 1**	Negative	83	15				
	Positive	0	84				

**Table 3:** Overall agreement (left part) and kappa coefficient with 95%CI (right part) comparing positive and negative results between methods.

	Farr-FIA	CLIA	CLIFT 1	CLIFT 2
**Farr-FIA**		0.714	0.730	0.582
		(95% CI: 0.612 to 0.817)	(95% CI: 0.632 to 0.828)	(95% CI: 0.475 to 0.690)
**CLIA**	86.3%		0.656	0.631
			(95% CI: 0.546 to 0.766)	(95% CI: 0.521 to 0.740)
**CLIFT 1**	86.8%	83.0%		0.836
				(95% CI: 0.758 to 0.915)
**CLIFT 2**	78.6%	81.3%	91.8%	

Legend: Green indicates the highest agreement/kappa coefficient, red demonstrates the lowest agreement/kappa coefficient, and the color gradient between green and red represents progressively decreasing levels of agreement/kappa coefficient.

**Table 4:** Overall agreement (left part) and kappa coefficient with 95%CI (right part) comparing positive and negative results between diagnostic combinations.

	CLIFT 1 and Farr-FIA	CLIFT 2 and Farr-FIA	CLIA and Farr-FIA	CLIA and CLIFT 1	CLIA and CLIFT 2
**CLIFT 1 and** **Farr-FIA**		1.000	0.938	0.855	0.788
			(95% CI: 0.885 to 0.992)	(95% CI: 0.777 to 0.934)	(95% CI: 0.696 to 0.880)
**CLIFT 2 and** **Farr-FIA**	100%		0.938	0.855	0.788
			(95% CI: 0.885 to 0.992)	(95% CI: 0.777 to 0.934)	(95% CI: 0.696 to 0.880)
**CLIA and** **Farr-FIA**	97.3%	97.3%		0.891	0.822
				(95% CI: 0.821 to 0.960)	(95% CI: 0.737 to 0.907)
**CLIA and** **CLIFT 1**	93.4%	93.4%	95.1%		0.930
					(95% CI: 0.875 to 0.985)
**CLIA and** **CLIFT 2**	90.1%	90.1%	91.8%	96.7%	

Legend: Green indicates the highest agreement/kappa coefficient, red demonstrates the lowest agreement/kappa coefficient, and the color gradient between green and red represents progressively decreasing levels of agreement/kappa coefficient.

## Data Availability

The datasets generated and analyzed during the current study are available from the corresponding author to ensure patient confidentiality regarding the included clinical diagnoses.
